# 异基因造血干细胞移植患者合并结核分枝杆菌感染诊断与治疗中国专家共识（2023年版）

**DOI:** 10.3760/cma.j.issn.0253-2727.2023.02.003

**Published:** 2023-02

**Authors:** 中华医学会血液学分会 中华医学会结核病学分会

结核分枝杆菌（mycobacterium tuberculosis，MTB）感染可表现为潜伏结核感染（latent tuberculosis infection，LTBI）和活动性结核病（active tuberculosis，ATB）。异基因造血干细胞移植（allogeneic hematopoietic stem cell transplantation，allo-HSCT）是MTB感染的独立危险因素，allo-HSCT患者LTBI进展为ATB的风险高于一般人群[1]–[2]。由于机体免疫状态的改变，allo-HSCT患者合并ATB的临床表现和辅助检查可不典型，临床诊断困难。抗结核药物的毒性及其与预处理药物、免疫抑制剂等多种药物之间的相互作用，使allo-HSCT合并ATB的防治更为复杂与困难。ATB是一种严重危害移植患者生存的合并症[3]–[4]。由中华医学会血液学分会和结核病学分会专家结合我国实际情况和防治现状联合撰写本共识，旨在促进allo-HSCT合并MTB感染的诊治规范化，为临床实践提供依据。

一、allo-HSCT患者ATB的流行病学特征

1. allo-HSCT患者MTB感染发生率与死亡率：中国是MTB感染发生率较高的国家之一，根据WHO 2022年公布的数据，2021年，中国结核病发生率约为55/10万，占全球总病例的7.4％[5]。移植相关免疫抑制剂的使用显著增加了LTBI进展为ATB的风险，HSCT患者ATB发生率是普通人群的10～40倍，为0.1％～16％[1]–[2],[6]–[9]。国内单中心数据显示allo-HSCT后ATB发生率约0.5％，是普通人群的10倍左右[9]。

HSCT患者合并ATB的死亡率较高，与移植类型、免疫抑制程度、诊断治疗是否及时、患者年龄、合并症及治疗反应等有关[3]–[4],[10]–[12]。播散性ATB进展迅速，可出现高热、成人呼吸窘迫综合征、低氧血症、脓毒症、多脏器功能衰竭，死亡率较高[12]–[13]。

2. allo-HSCT患者ATB的危险因素：HSCT患者合并ATB主要来源于移植前内源性复燃及外源性再感染。既往有MTB感染或密切接触史是HSCT患者发生ATB的主要危险因素[9]，其他危险因素还有：血液系统恶性疾病、同种异基因移植物去除T细胞、人类白细胞抗原（human leukocyte antigen, HLA）无关供者移植、亲缘HLA不全相合移植、急性和慢性移植物抗宿主病、侵袭性真菌感染、应用某些药物（如糖皮质激素、他克莫司、芦可替尼等）[1],[14]–[19]。

二、allo-HSCT前MTB感染的筛查与评估

1. allo-HSCT受者MTB感染的筛查与评估：为了降低allo-HSCT患者合并ATB的发生率，对拟行HSCT的患者进行MTB感染筛查极为重要。建议所有接受allo-HSCT的患者均应常规进行LTBI筛查，并评估有无ATB，常需与呼吸、感染、结核等专科进行多学科协作。

筛查与评估的主要内容包括：①既往MTB感染病史；②活动性肺结核患者密切接触史；③有无ATB相关临床症状：如咳嗽、咳痰、咯血等呼吸道症状和低热、盗汗、体重下降等全身症状；④细胞免疫学检测：γ干扰素释放试验（tuberculosis interferon gamma release assay, IGRA）、重组结核杆菌融合蛋白（EC）皮肤试验和结核菌素皮肤试验（tuberculin skin test, TST）；⑤影像学检查：胸部高分辨CT和经临床医师判断需要检查的其他部位的影像学检查；⑥对免疫学检测阳性和（或）具有结核相关影像学表现者，进行痰涂片、痰培养，以及相应标本的MTB分子生物学检测等，以排除ATB。

移植前如发现LTBI，并排除ATB，建议在移植期间进行预防性抗结核治疗，不必因此推迟allo-HSCT[1]–[2],[20]–[21]。移植前如诊断ATB，应开始抗结核治疗，并推迟allo-HSCT至临床判断活动性感染得到良好控制方可进行。

仅胸部高分辨CT发现多发微结节而非典型肺结核影像学时应高度警惕并加强HSCT后的动态评估，尤其是对于高龄和低外周淋巴细胞计数的患者。

2. allo-HSCT供者MTB感染的筛查与评估：在捐献干细胞之前应对供者进行MTB感染的筛查与评估，方法同受者[21]。

建议有ATB的供者应延迟捐献干细胞直至ATB得到良好控制，或更换供者。

三、allo-HSCT患者LTBI的诊断与预防性治疗

1. LTBI概念：LTBI是指机体对MTB抗原刺激产生持续的免疫应答但无临床活动性结核病的一种状态。LTBI者没有结核感染的症状与体征，没有病原学、病理学、影像学的活动性结核感染的证据。LTBI不具有传染性，但机体免疫状态发生改变后可进展为ATB，是否进展为ATB与机体的免疫状态密切相关[1],[9],[20]。

2. LTBI检测：目前尚无用于LTBI检测的“金标准”。临床上用于LTBI检测的主要方法有IGRA（包括T-SPOT.TB及QFT-GIT）、EC皮肤试验和TST[1],[20],[22]。TST简单易行，但无法排除卡介苗（BCG）接种的影响，在免疫缺陷人群中的灵敏度较低[1],[22]。EC皮肤试验又称新型结核菌素皮肤实验（creation tuberculosis skin test, C-TST），EC包含针对MTB的特异性抗原EAST-6和CFP-10，在BCG和其他大多数非结核分枝杆菌（nontuberculosis mycobacteria, NTM）中不含这些抗原，因此，该试验可以有效鉴别BCG接种和MTB感染[20]。IGRA的特异性优于TST，因其与BCG和NTM的交叉反应较少[1],[20],[22]。

在allo-HSCT受者中，IGRA的不确定结果比例相对较高，IGRA假阴性可见于免疫功能低下、接受免疫抑制剂治疗、高龄、外周血低淋巴细胞计数等情况[2],[23]。目前IGRA主要是基于外周血检测，其在体液标本的敏感性和特异性欠佳，且不同部位的IGRA性能有差异[23]。胸腔积液的IGRA反应水平比外周血高，设立判断阈值时不能以外周血的判断水平作为参考，需要各实验室根据自身情况设定阈值。腹腔积液及心包积液的操作及临床意义参考胸腔积液，对于其他如脑脊液、关节液、肺泡灌洗液等标本，由于很难分离到足够数量的淋巴细胞，IGRA敏感性和特异性均明显降低[23]。

推荐意见：

（1）对所有拟行allo-HSCT的患者进行LTBI筛查，并排除ATB。

（2）IGRA和EC皮肤试验作为allo-HSCT患者LTBI筛查的首选方法，IGRA和EC皮肤试验不可获得或无法进行时行TST[1]。

（3）IGRA、EC皮肤试验或TST阳性者考虑为LTBI。

（4）如果IGRA结果为不确定值（介于阳性-阴性之间），不建议重复检测IGRA[1]。

（5）对于活动性肺结核感染密切接触史人群，如果初始细胞免疫学检测阴性，可在最后一次接触的8～12周后重复检测[1]。如果患者有疑似ATB的临床症状和体征，即使细胞免疫学阴性，仍需进一步筛查ATB。

（6）移植前细胞免疫学阴性受者并非“零风险”，仍有较低的风险出现ATB[24]–[25]。

（7）胸腔积液的IGRA检测流程及阈值还需进一步探讨与规范，对于证据不足的肺外结核患者，胸腔积液和外周血IGRA检测同时进行，有一定补充或辅助性诊断的作用，各实验室需重新验证胸腔积液IGRA的最佳阈值。其他体液标本如腹腔积液、心包积液、脑脊液等临床样本的IGRA检测缺乏统一操作流程和判读标准。

3. 预防性抗结核治疗：预防性抗结核治疗是指对LTBI的患者给予治疗措施以减少进展为ATB的风险。开始预防性抗结核治疗同时不必因此推迟allo-HSCT进行[1],[21]。

推荐对以下allo-HSCT人群考虑进行预防性抗结核治疗，常需与呼吸、感染、结核等多学科协作制定具体方案，并排除预防性抗结核治疗禁忌证：①诊断LTBI，且排除ATB[1]–[2],[21]；②既往ATB病史，经规范化治疗结束后超过5年。

预防治疗方案：①首选异烟肼，剂量为5～10 mg·kg^−1^·d^−1^，至少应用6～9个月，持续到免疫抑制剂减量。异烟肼开始预防的时机可从预处理或预处理前开始。异烟肼在HSCT后的安全性和耐受性较好，可与氟康唑、伏立康唑、泊沙康唑合并用药，不推荐与伊曲康唑同用，治疗期间应监测肝功能[26]。②利福平谨慎应用，因为利福平与免疫抑制剂和其他药物之间可能存在复杂的药物相互作用，在allo-HSCT受者中应考虑合并用药间的相互作用[27]。③如果有明确传染源且传染源确诊为耐利福平或异烟肼，则抗结核治疗方案应由结核病专家组根据传染源的耐药谱制定，并需做详细的风险评估和治疗方案论证。

四、allo-HSCT患者ATB的诊断

allo-HSCT患者合并ATB的诊断是以病原学（包括涂片、培养、分子生物学）检测和病理学检查为主要依据，结合流行病史、临床表现、影像、相关的辅助检查及鉴别诊断等，进行综合分析做出诊断。HSCT患者ATB的临床表现及诊断有其特殊性，不能简单完全套用普通人群诊断标准。在进行诊断时，应注意患者的免疫功能状态对患者的临床表现及结核病诊断技术的灵敏性与特异性均可能存在一定影响。

1. 定义：参考WHO指南和《中华人民共和国卫生行业标准肺结核诊断》[22],[28]–[29]，ATB是指存在临床症状和体征，以及结核病的病理、细菌学和（或）影像学证据。结核诊断包括疑似病例、临床诊断病例和确诊病例[30]。

根据感染的解剖部位分为肺结核（pulmonary tuberculosis, PTB）和肺外结核（extrapulmonary tuberculosis, EPTB）。粟粒性肺结核和结核性胸膜炎因在肺部或胸膜有病变，被归类为PTB。同时存在肺结核和肺外结核的患者应归为PTB。

按照是否耐药将结核病分为敏感结核病和耐药结核病。耐药结核病（drug-resistant tuberculosis, DR-TB）根据体外临床分离株的药敏试验（drug susceptibility testing, DST）或者分子药敏进行分类：①单耐药结核：结核分枝杆菌对一种一线抗结核药物耐药；②多耐药结核：结核分枝杆菌对一种以上一线抗结核药物耐药，但不包括异烟肼、利福平同时耐药；③耐多药结核（multidrug-resistant tuberculosis, MDR-TB）：结核分枝杆菌对至少两种以上的一线抗结核药物耐药，包括异烟肼、利福平同时耐药；④广泛耐药结核（extensively drug-resistant tuberculosis, XDR-TB）：结核分枝杆菌除对一线抗结核药物异烟肼、利福平同时耐药外，还对二线抗结核药物氟喹诺酮类抗生素中的至少一种以及贝达喹啉和利奈唑胺至少一种耐药；⑤利福平耐药结核（rifampicin-resistant tuberculosis，RR-TB）：结核分枝杆菌对利福平耐药，无论是否对其他抗结核药物耐药[22]。

2. 临床表现：咳嗽、咳痰≥2周，或痰中带血或咯血为肺结核可疑症状。结核的全身症状，如发热、盗汗和体重下降，在移植受者中并无特异性，对于未明确病原体的发热，要高度怀疑MTB感染的可能，并进行相应的检查。allo-HSCT后ATB诊断的中位时间是移植后8个月左右，范围从移植后数周至数年不等[3]–[4],[9],[15],[31]–[32]。肺部是最常感染的器官，约存在于四分之三的感染病例[4],[33]–[34]。肺结核可能与侵袭性真菌感染共存，如毛霉菌病或曲霉菌病。在这些患者中结核的表现可类似侵袭性真菌感染，甚至出现结核分枝杆菌菌血症，并迅速进展[10]–[11],[35]–[36]。至少三分之一HSCT后ATB病例伴有肺外播散，部位包括肝、脾、骨、骨髓、脑和脊柱等[12],[37]–[39]。中枢神经系统感染可能以脑膜炎或颅内占位性病变的形式出现，结核病腹膜炎可表现为急腹症，腹部肿块压迫和肠粘连可引起肠梗阻[12],[37]–[39]。

3. MTB检查：痰液、支气管冲洗液或支气管肺泡灌洗液、经支气管肺活检、脑脊液、尿液、肺结核和肺外结核病变处组织活检标本等进行分枝杆菌检测并除外非结核分枝杆菌是诊断HSCT后ATB最为直接的证据。MTB检查方法主要有涂片镜检、培养和分子生物学检测。

（1）涂片镜检和培养：涂片和培养是目前临床诊断ATB的基本检测方法，也是评价抗结核疗效的主要方法。但镜检和培养的阳性率和分离率相对较低且受标本质量的影响。

推荐意见：

送检抗酸杆菌染色涂片镜检和分枝杆菌培养，建议所有标本在液体和固体培养基中培养8～12周，以提高阳性率和培养分离率。培养出的菌落通过分子或表型方法（MALDI-TOF光谱）药敏并除外非结核分枝杆菌，如初筛为非结核分枝杆菌应在群、种或亚种水平上进行鉴定。对分离出的结核分枝杆菌进行DST[40]。如果该菌株对一线药物（如异烟肼、利福平、乙胺丁醇和吡嗪酰胺）敏感，则不必须进行二线药物DST，除非该方案不使用利福平。对于耐多药（MDR）分离株应进行综合基因型检测和DST[22],[40]。

（2）分子诊断：

推荐意见：

分子诊断灵敏度高于痰涂片和培养，对疑似结核病患者进行至少1次相关临床标本的MTB核酸检测。Xpert MTB/RIF或者Xpert MTB/RIF Ultra可作为疑似结核病的初始诊断和利福平耐药检测方法，但不能用于抗结核疗效的评价[22],[41]。

其他新型检测方法，如宏基因组二代测序技术（mNGS）在TB诊断中显示出较好的特异性，建议在临床疑似，而采用传统培养和分子生物学方法诊断困难的病例，尤其是肺外结核病例中考虑应用。

尽可能尽早留取标本来明确诊断。对于肺结核的诊断，进行痰抗酸染色涂片镜检、痰培养和痰MTB快速分子学检测，有条件时应同时送检支气管吸取物或肺泡灌洗液进行病原学检测。对于肺外结核留取相应标本，如脑脊液、胸腔积液、腹腔积液、心包积液、粪便、尿液等进行抗酸染色涂片镜检、培养和MTB快速分子生物学检测。血培养阳性率极低，但有条件时应尽可能进行，但对于血培养分枝杆菌阳性的患者，应排除非结核分枝杆菌感染的可能。

4. 结核病病理学检查：结核典型的病理学改变表现为干酪样肉芽肿，光学显微镜下可见大小不等和数量不同的坏死性和非坏死性的肉芽肿。免疫抑制人群典型结核性肉芽肿可表现为形成不良甚至缺乏。淋巴结穿刺标本行病理学检查及涂片和培养对于结核病的诊断价值较高。典型结核的病理诊断较容易但仍需要MTB检测阳性，多数结核病灶特别是干酪样坏死组织中及其周围组织内可查到抗酸杆菌，还需要采用现代分子生物学检测手段，如聚合酶链反应（PCR法）、原位杂交和基因测序等作出诊断。

5. 细胞免疫学检查：对于少数诊断困难的ATB尤其是肺外结核，可进行细胞免疫学检查以协助诊断，但细胞免疫学检查不能区分LTBI和ATB。因IGRA在allo-HSCT人群中敏感性较低，需注意阴性检测结果也不能完全除ATB的可能。

6. 影像学检查：影像学检查对于HSCT患者结核病的筛查和诊断具有重要价值，首选胸部高分辨CT平扫检查。由于肺结核最为常见，所以胸部高分辨CT推荐常规进行[30]。免疫抑制人群的肺结核影像学表现可与普通人群不同：肺下叶、中叶间质性和粟粒样渗出多见，而空洞性病变少见。

五、HSCT患者合并活动性结核病的治疗

原则上，HSCT患者结核病的管理与治疗和单纯结核患者相同，按照《中国结核病预防控制工作指南》进行管理，但需注意：①抗结核药物和免疫抑制剂、预处理药物等多种药物间的相互作用；②抗结核治疗与原发病治疗之间的权衡。其治疗常需与结核、感染、呼吸等多学科协作。

1. 药物敏感ATB的治疗：

（1）推荐对allo-HSCT患者的药物敏感ATB使用与当地非HSCT患者相同的结核治疗方案：通常为6个月短程化疗，建议每日服药，而不主张采取间歇治疗[42]–[43]。然而，该方案尚未在HSCT患者中进行充分评价，临床上需对此问题进行进一步研究。常用药物敏感抗结核药物的建议剂量及用法见表1。

**表1:** 常用一线抗结核药物建议剂量及用法（成人）

药物	建议剂量（最大剂量）	用法
异烟肼	5～10 mg·kg^−1^·d^−1^（300 mg/d）	口服
利福平	10 mg·kg^−1^·d^−1^（600 mg/d）	口服
利福喷丁	450～600 mg，每周2次	口服
利福布汀	5 mg·kg^−1^·d^−1^（300 mg/d）	口服
吡嗪酰胺	15～30 mg·kg^−1^·d^−1^（2 000 mg/d）	口服
乙胺丁醇	15～25 mg·kg^−1^·d^−1^	口服

（2）考虑到利福霉素类抗结核药物与免疫抑制剂之间的代谢干扰，对于正在使用钙神经蛋白抑制剂（calcineurin inhibitor, CNI）和哺乳动物雷帕霉素靶蛋白抑制（mammalian target of rapamycin inhibitor, mTORi）的allo-HSCT患者，建议避免应用利福平。与利福平相比，利福喷丁和利福布汀的细胞色素P450诱导作用较弱，与这些药物的相互作用要小得多，因此建议移植后患者使用利福喷丁或利福布汀代替利福平；正在应用免疫抑制剂的HSCT受者接受利福平治疗时需要适当增加免疫抑制剂的剂量，皮质类固醇剂量需增加1倍，并监测CNI和mTORi血药浓度。常用抗结核药物与免疫抑制剂的相互作用见表2。

**表2:** 常用抗结核药物与免疫抑制剂间的相互作用

药物	异烟肼	利福平	吡嗪酰胺	乙胺丁醇	链霉素	莫西沙星或左氧氟沙星
环孢素A	无影响	降低环孢素A血药浓度及疗效（肝代谢诱导）	无影响	无影响	增加肾毒性的风险	增加环孢素A血药浓度（仅左氧氟沙星）
他克莫司	无影响	降低他克莫司血药浓度及疗效（肝代谢诱导）	无影响	无影响	增加肾毒性的风险	无影响
西罗莫司	无影响	降低西罗莫司血药浓度及疗效（肝代谢诱导）	无影响	无影响	无影响	无影响
吗替麦考酚酯	无影响	降低吗替麦考酚酯血药浓度及疗效（肠肝循环障碍）	无影响	无影响	无影响	降低吗替麦考酚酯的血药浓度

（3）对于结核病病情较轻和免疫功能较差的患者（如慢性移植物抗宿主病），可选用不联合利福霉素类的抗结核方案，从而降低移植排斥反应发生的风险，无利福霉素方案所需疗程更长（至少9～12个月）[18],[33]。

（4）疗程长短还需参考对抗结核治疗的反应、用药方案和宿主免疫状态。

（5）针对不同感染部位肺外结核推荐的抗结核疗程：骨、关节结核治疗疗程为至少12个月；中枢神经系统结核为至少12～18个月；血行播散结核为至少12个月[44]。

（6）抗结核治疗后的疗效评估和随访与非HSCT患者相同，由于患者同时接受原发病治疗和（或）免疫抑制治疗，建议进行更为密切的随访和观察。每月应对患者服用抗结核药物的耐受性、依从性、疗效及不良反应进行评价，应采取措施提高患者服药的依从性。必要时可借助治疗药物浓度监测技术来指导和调整治疗方案。

2. MDR/XDR-TB的治疗：

（1）MDR-TB和XDR-TB的治疗应该个体化，应参考WHO耐药结核病指南[45]、中国防痨协会和中华医学会结核病学分会制定的相关指南和共识来制定结核治疗方案[46]–[47]。建议采用长疗程多药联合方案（4种及以上），但以上指南缺乏针对HSCT受者的推荐意见，因此，建议与结核、感染、呼吸等多学科协作[45]–[47]。

（2）MDR-TB和XDR-TB的治疗必须依赖于全面的表型和基因型药敏试验，需要更长时间的治疗（18～20个月）。

3. 诊断性治疗：

（1）对于临床上高度怀疑ATB的患者，在系统检查后仍无法明确诊断，并除外其他可能的肺部疾病患者，可考虑进行诊断性治疗，但需严格掌握指征并客观评估诊断性治疗的反应。建议诊断性治疗为4联抗结核药物的标准治疗方案，2个月后再行进一步评估及诊断。

（2）暂时不能确诊而疑似其他致病菌感染的患者，可进行抗感染治疗（一般观察2周）或使用其他检查方法进一步确诊，抗感染治疗不能使用喹诺酮类、氨基糖苷类等具有明显抗结核作用的药品。

4. 其他治疗糖皮质激素有助于提高结核性脑膜炎患者的生存率，合并结核性脑膜炎治疗中建议应用糖皮质激素，但目前尚无研究比较不同剂量和疗程的糖皮质激素使用的疗效和不良反应差异，因此，糖皮质激素使用的最佳剂量和疗程尚待研究[43],[48]。

根据WHO药物敏感结核治疗指南，结核性心包炎患者可使用糖皮质激素[43]，建议在一些情况下如大量心包积液及心包穿刺液中炎症标志物或炎症细胞升高明显的则可酌情考虑使用糖皮质激素，对于积液量少于5 mm的积液-缩窄和缩窄性结核性心包炎慢性患者，不推荐应用糖皮质激素[48]–[49]。

allo-HSCT合并ATB患者出现抑郁状态、焦虑心理状态的风险较大，心理状态可能影响患者治疗依从性，从而影响治疗结局，在抗结核治疗过程中关注患者的心理状态，加强社会支持[50]–[51]。

六、结语

ATB是allo-HSCT后少见但严重的并发症，allo-HSCT后ATB的诊断和防治面临许多挑战，本共识基于我国循证医学证据，参考国际相关指南，明确了allo-HSCT患者结核感染的筛查、结核病预防、诊断与治疗原则，旨在更好规范allo-HSCT合并MTB感染管理策略，进一步助力提升我国在allo-HSCT后MTB感染管理的临床诊疗水平，改善患者长期生存。
